# Cardiogenic shock and acute renal failure associated mortality trends in the United States: a retrospective analysis of death records from 1999 to 2023

**DOI:** 10.1186/s12872-026-05698-9

**Published:** 2026-03-04

**Authors:** Muhammad Shaheer Bin Faheem, Syed Tawassul Hassan, Syeda Lyba Onaiz, Anushah Faheem, Shan e Ali, Naveed Ahmad, Sumaya Samadi

**Affiliations:** 1 Karachi Institute of Medical Sciences, KIMS, Karachi, Sindh Pakistan; 2 Karachi Medical and Dental College, KMDC, Karachi, Sindh Pakistan; 3 Foundation University Medical College, FUMC, Islamabad, Pakistan; 4 Jinnah Sindh Medical University, JSMU, Karachi, Sindh Pakistan; 5 Khyber Medical College, Peshawar, Khyber Pakhtunkhwa Pakistan; 6 Kabul University of Medical Sciences “Abu Ali Ibn Sina”, Kabul, Afghanistan

**Keywords:** Cardiogenic Shock, Acute Renal Failure, Mortality Trends, Health Disparities, CDC WONDER

## Abstract

**Background:**

Cardiogenic shock (CS) and Acute Renal Failure (ARF) have a compounding impact on patient survival. The long-term epidemiological burden of this cardiorenal comorbidity remains understudied despite the advances in mechanical circulatory support and critical care. We aimed to analyze nationwide mortality trends and disparities involving co-occurring CS and ARF in the United States.

**Methods:**

Death records from the CDC WONDER database were analyzed retrospectively (1999–2023), including adults aged ≥ 45 years listing CS and ARF as underlying or contributing causes. Age-Adjusted Mortality Rates (AAMRs) per 1,000,000 population were standardized to the 2000 U.S. population. Temporal trend assessment and annual percent changes (APCs) were computed through the Joinpoint regression program.

**Results:**

A total of 48,926 deaths were identified with AAMR, increasing significantly from 7.0 in 1999 to 43.5 in 2023. Rates showed a steady incline from 2005 to 2019 (APC: 7.78), followed by a sharp acceleration from 2019 to 2023 (APC: 24.16). Males exhibited mortality rates nearly double those of females (20.5 vs. 10.5). Non-Hispanic (NH) African Americans had the highest AAMR (20.7) among racial groups. Geographically, the West (17.1) and non-metropolitan regions (12.9) experienced the highest mortality burdens.

**Conclusions:**

The past two decades marked the surge in CS and ARF-related mortality rates following a sharp acceleration since 2019. Persistent disparities among males, NH African Americans, and rural populations highlight the urgent need for targeted resource allocation and improved access to advanced cardiorenal therapies.

**Clinical trial number:**

Not applicable.

**Supplementary Information:**

The online version contains supplementary material available at 10.1186/s12872-026-05698-9.

## Introduction

Cardiogenic shock (CS) is a deadly syndrome caused by inadequate cardiac output as well as hypoperfusion of end-organs, leading most commonly to multi-organ dysfunction with high mortality even in the era of advanced cardiovascular care [1, 2]. Contemporary management increasingly involves percutaneous mechanical circulatory support devices such as Impella, intra-aortic balloon pump (IABP), and VA-ECMO (Veno-Arterial Extracorporeal Membrane Oxygenation), which have revolutionized clinical practice and outcomes for particular patient populations [1]. Vasopressors and inotropes are still essentially required to stabilize hemodynamics in acute myocardial infarction (AMI) related CS; however, evidence has indicated that their careful titration influences short-term survival as well as renal perfusion [3]. CS complicates approximately 5–10% of cases of AMI, with in-hospital mortality rates wherein the availability of modern revascularization and mechanical circulatory support does not bring the rate below 40% [4, 5]. Worst outcomes among older adults and patients specifically with pre-existing heart failure (HF) raise a significant healthcare burden, highlighting the need for improved management strategies [6, 7]. Longitudinal analyses underscore the fact that age-adjusted mortality rates (AAMRs) related to CS have increased over the last two decades, highlighting the persistent public health challenge [5].

Acute Renal Failure (ARF) is a common and serious complication in patients with CS resulting from renal hypoperfusion, venous congestion, systemic inflammation, and neurohormonal dysregulation [8, 9]. Clinical studies have indicated that ARF develops in about 40–50% of patients with CS. It has an independent association with increased short-term and in-hospital mortality [6, 8]. Furthermore, mortality risk has also been significantly impacted among VA-ECMO-supported CS patients who have also developed ARF [10]. The severity of ARF by KDIGO (Kidney Disease: Improving Global Outcomes) classification showed a dose-dependent relationship with mortality, supporting the notion that kidney injury has a great specific pathophysiological role, not simply reflecting the general severity of illness [7, 8]. Therefore, long-term adverse events in survivors after recovering from CS are complicated by ARF, including progression to chronic kidney disease, rehospitalization, and major cardiovascular events, which are also precipitated [11, 12].

Most former studies focused either on CS or ARF independently and highlighted the causes and effects of CS related ARF. They lack national-level data regarding the long-term mortality burden posed by these concurrent conditions across demographics and geographics. The Centers for Disease Control and Prevention Wide-ranging Online Data for Epidemiologic Research (CDC WONDER) database provides a unique opportunity to assess these trends and disparities. Methodological studies have validated the utility of Multiple Cause of Death (MCD) data for analyzing complex, multi-cause mortality scenarios over extensive periods, allowing for the identification of high-risk subgroups across age, sex, and race [13, 14]. Population-level data integrated with clinical registries will surface high-risk subgroups that are of the older adult population, racial/ethnic minority groups, and patients who suffer from pre-existing comorbid conditions for targeted public health interventions [11, 15].

Therefore, this paper uses CDC WONDER-MCD data from 1999 to 2023 to analyze co-existing CS and ARF mortality trends among adults (aged ≥ 45 years) in the U.S. and identifies the demographic and geographic mortality disparities. We aim to provide population-level evidence to guide policy-making bodies to take necessary steps for adequate resource allocation and interventions across populations with increased mortality burden [1, 2, 5, 6, 13].

## Methods

### Study parameters and population

This study utilized data from the CDC WONDER database to examine mortality trends associated with CS and ARF in the U.S. from 1999 to 2023 [16]. The Multiple Cause-of-Death Public Use record death certificates were analyzed to identify deaths in which both CS and ARF were listed as either the underlying or contributing cause of death. CS was identified using the International Classification of Diseases, Tenth Revision, Clinical Modification (ICD-10-CM) code R57.0, while ARF was identified using ICD-10-CM code N17 [17]. The study population included adults aged ≥ 45 years. These ICD-10 definitions have been widely used in prior epidemiological studies evaluating cardiovascular and renal-related mortality trends. Institutional Review Board (IRB) approval and informed consent were not required because the database contains de-identified, publicly available data. This study adhered to the Strengthening the Reporting of Observational Studies in Epidemiology (STROBE) guidelines [18].

### Data sourcing

All deaths among individuals aged ≥ 45 years meeting the specified ICD-10 criteria were included. Mortality data were stratified by sex, race/ethnicity, age, U.S. census region, state of residence, and level of urbanization. Race and ethnicity were categorized as non-Hispanic (NH) White, NH African American, and Hispanic populations. County-level urbanization status was classified as urban or rural according to the National Center for Health Statistics (NCHS) urban-rural classification scheme [19].

### Statistical analysis

AAMRs per 1000,000 population associated with CS and ARF were obtained from the CDC WONDER database, along with corresponding 95% confidence intervals (CIs). Age adjustment was performed using the direct standardization method based on the 2000 U.S. standard population to account for differences in age distribution over time [20]. Temporal trends in AAMRs from 1999 to 2023 were assessed using the Joinpoint Regression Program (version 5.1.0, National Cancer Institute) [21]. Joinpoint regression models identify statistically significant changes in mortality trends over time by fitting log-linear regression segments connected at joinpoints. Further, the program inherently smooths out the isolated single-year mortality surges and incorporates these short-term anomalies into the variance of the model instead of distorting the broader temporal trajectory represented by the annual percent change (APC). The APC was calculated to describe mortality trends over the study period. Statistical significance was determined using a two-sided t-test, with a p-value < 0.05 considered statistically significant. Further, we performed a sensitivity analysis to account for the temporal ambiguity inherent in death certificates. For this, we restricted our population cohort to deaths where ARF was explicitly coded as the underlying cause and CS as a contributing cause of death.

## Results

### Proportional mortality rate across subgroups

From 1999 to 2023, a total of 48,926 deaths were attributed to CS and ARF in the U.S. The highest fatalities were reported in males (60.23%), NH Whites (72.60%), and older individuals aged ≥ 65years (77.11%). Further medical facilities (97%), urban areas (81.02%), and the south region (40.15%) represented the largest proportion of mortality. The proportional mortality data reflect the distribution of the number of deaths as opposed to the actual rates of death, which will be discussed in trend analysis subsequently (Supplementary Table 1, Supplementary Fig. 1).

### Overall and sex stratified trends

**Fig. 1:**
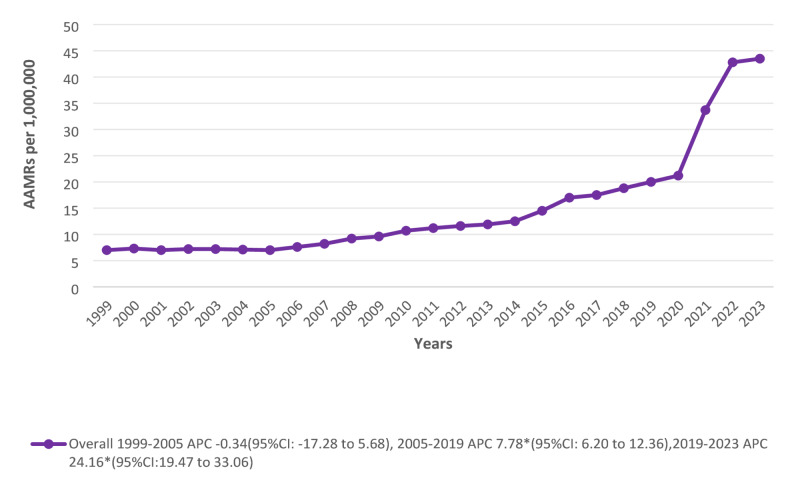
Overall trends in Cardiogenic Shock and Acute renal failure-related age-adjusted mortality rates per 1,000,000 in the United States, 1999 to 2023. APC = Annual Percentage Change, CI = Confidence Interval, AAMRs = Age-adjusted Mortality Rate

**Fig. 2:**
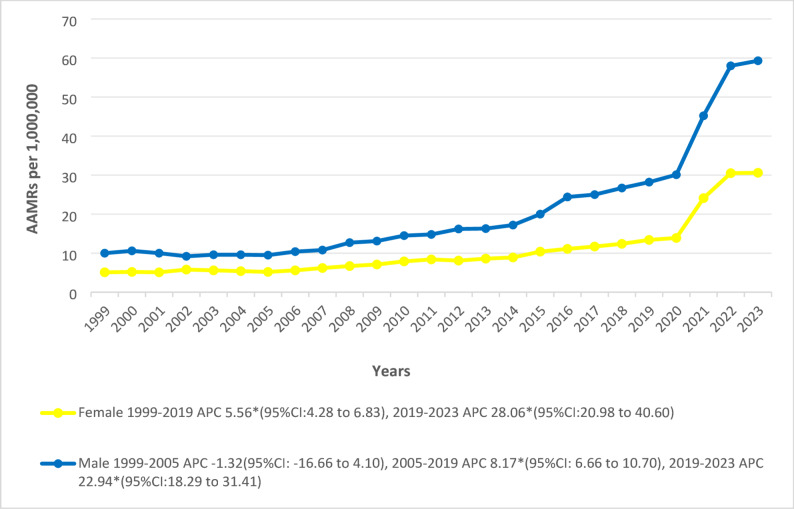
Trends in Cardiogenic Shock and Acute renal failure-related age-adjusted mortality rates per 1,000,000, stratified by sex in the United States, 1999 to 2023. APC = Annual Percentage Change, CI = Confidence Interval, AAMRs = Age-adjusted Mortality Rate. *Indicates that the Annual Percentage Change (APC) is significantly different from zero at α = 0.05

The overall AAMR per 1,000,000 U.S. population significantly escalated from 1999 (7.00) to 2023 (43.5). Initially, mortality rates followed a stabilized non-significant trend till 2005, after which they started a sustained statistically significant uptrend till 2019 (APC: 7.78*; 95% CI: 6.20 to 12.36) followed by a more pronounced increase through 2023 (APC: 24.16*; 95% CI: 19.47 to 33.06). Males exhibited almost twice the total average mortality rate as females (20.5 vs. 10.5). Males AAMR showed a period of non-significant decline from 1999 to 2005, followed by sharp surges between 2005 and 2019 and 2019–2023 with APCs of 8.17* (95% CI: 6.66 to 10.70) and 22.94* (95% CI:18.29 to 31.41) respectively. However, a significant incline was observed in females from 1999 to 2019, followed by another spike through 2023 with associated APCs of 5.56* (95% CI: 4.28 to 6.83) and 28.06* (95% CI: 20.98 to 40.60), respectively (Figs. 1 and 2) (Supplementary Tables 2, 3). 

### Sensitivity analysis for temporal bias

Death certificates listing both conditions may not necessarily establish a temporal sequence, and it remains unclear whether CS incited ARF or pre-existing ARF exacerbated CS. To account for uncertainty, we conducted a sensitivity analysis by limiting the query to deaths where ARF was strictly the underlying cause of death and CS was a contributing consequence (*n* = 7,872). We observed a similar pattern as that of our primary analysis, and AAMRs of this subgroup rose steadily from 1999 (1.0) to 2019 (3.5), followed by a sharp significant acceleration through 2023 (5.6) with associated APCs of 6.44* (95% CI: 4.40 to 7.56) and 16.07* (95% CI: 9.25 to 30.49) respectively. However, this sensitivity analysis confirms the robustness of the rising mortality burden of this cardiorenal phenotype and eliminates the uncertainty regarding temporal coding bias (Supplemental Tables 1, 3, 4).

### Mortality trends across racial and ethnic subgroups

**Fig. 3:**
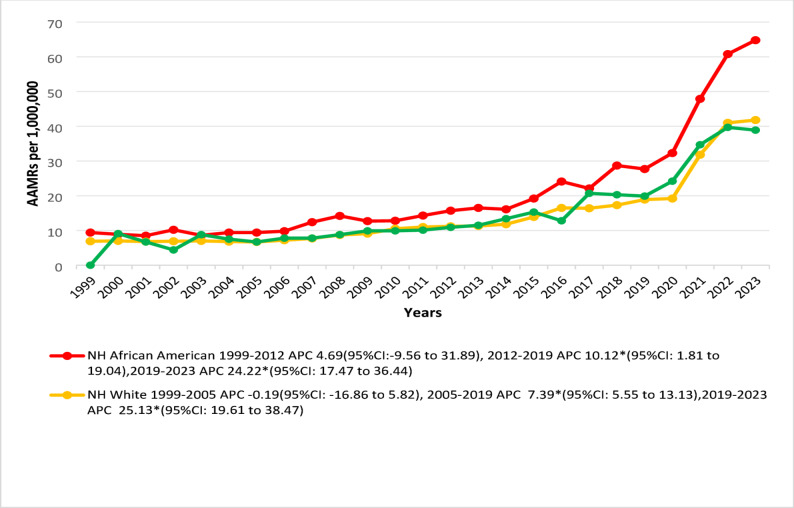
Trends in Cardiogenic Shock and Acute renal failure-related age-adjusted mortality rates per 1,000,000, stratified by race and ethnicity in the United States, 1999 to 2023. APC = Annual Percentage Change, CI = Confidence Interval, AAMRs = Age-adjusted Mortality Rate. *Indicates that the Annual Percentage Change (APC) is significantly different from zero at α = 0.05

The NH African Americans exhibited the highest total average AAMR (20.7), followed by Hispanics (15) and NH whites (14.1). NH Whites experienced an initial non-significant decrease in AAMR between 1999 and 2005, while NH African Americans and Hispanics exhibited non-significant increases during their respective early study periods. Following that, both NH African Americans and NH Whites experienced significant increases until 2019 and afterwards through 2023 with associated APCs of 10.12* (95% CI: 1.81 to 19.04) and 24.22* (95% CI: 17.47 to 36.44) in NH African Americans and 7.39* (95% CI: 5.55 to 13.13) and 25.13* (95% CI: 19.61 to 38.47) in NH Whites. However, Hispanics showed a significant increasing trend from 2013 to 2023 (APC 14.21*; 95% CI: 11.84 to 21.10) (Fig. 3; Supplementary Tables 3, 5).

### Age group analysis

**Fig. 4:**
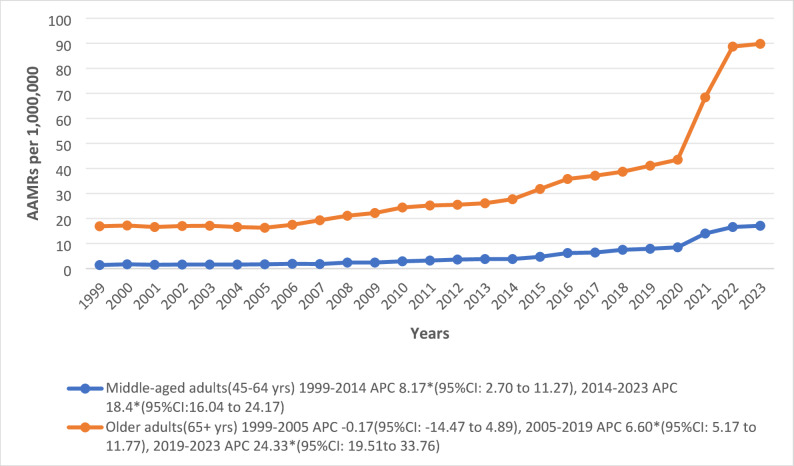
Trends in Cardiogenic Shock and Acute renal failure-related age-adjusted mortality rates per 1,000,000, stratified by Age groups in the United States, 1999 to 2023. APC = Annual Percentage Change, CI = Confidence Interval, AAMRs = Age-adjusted Mortality Rate. *Indicates that the Annual Percentage Change (APC) is significantly different from zero at α = 0.05

The individuals in the elderly cohort (32.1) who aged ≥ 65 years averaged sixfold higher than the AAMR of the middle-aged cohort (5.0) who aged 45–64 years. Significant surges were observed throughout the study among the individuals aged 45–64 years (APC: 1999–2014: 8.17*; 95% CI: 2.70 to 11.27; 2014–2023: 18.4*; 95% CI:16.04 to 24.17). In contrast, the elderly individuals (≥ 65 years) had an initial non-significant stable trend till 2005, followed by significant surges until 2019 (APC: 6.60*; 95% CI: 5.17 to 11.77) and through 2023 (APC: 24.33*; 95% CI: 19.51 to 33.76) (Fig. 4; Supplemental Tables 3, 6).

### Geographic trends

**Fig. 5:**
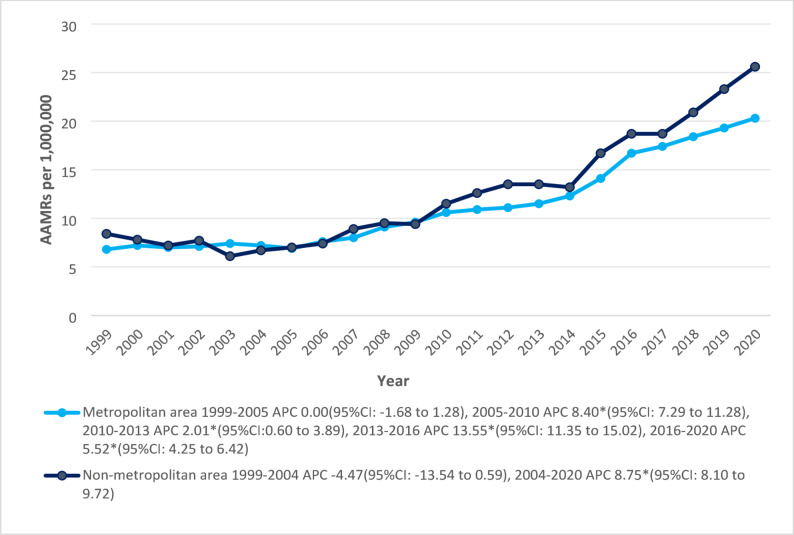
Trends in Cardiogenic Shock and Acute renal failure-related age-adjusted mortality rates per 1,000,000, stratified by urbanization in the United States, 1999 to 2020. APC = Annual Percentage Change, CI = Confidence Interval, AAMRs = Age-adjusted Mortality Rate. *Indicates that the Annual Percentage Change (APC) is significantly different from zero at α = 0.05

States (Indiana, Delaware, Washington, South Carolina, and Texas) in the upper 90th percentile had nearly three times higher rates than those of the bottom 10th percentile states (Alaska, New York, Montana, Vermont, and Colorado). The highest total average AAMR was observed in the West region (17.1), followed by the South (15.9), Midwest (13.4), and Northeast (11.5). Non-metropolitan or rural areas (12.9) had a higher AAMR than the metropolitan areas (11.8). Both metropolitan and non-metropolitan areas experienced a non-significant downward trend in the early 2000s. From there onwards, the metropolitan area demonstrated an escalating trend alternating in cycles of rapid rise between 2005 and 2010, 2010–2013, 2013–2016 and 2016–2020 with associated APCs of 8.40* (95% CI: 7.29 to 11.28), 2.01* (95% CI: 0.60 to 3.89), 13.55* (95%CI: 11.35 to 15.02) and 5.52* (95% CI: 4.25 to 6.42) respectively. In contrast, the non-metropolitan areas, after following a non-significant decrease until 2004, exhibited a substantial increase through 2020 (APC: 8.75*; 95%CI: 8.10 to 9.72) (Fig. 5) (Supplementary Fig. 2) (Supplemental Tables 3, 7–9).

### Etiological sub-classification

Further, an etiological sub-analysis was conducted to identify CS and ARF deaths specific to the underlying etiologies like AMI, HF, and cardiac arrhythmias, which may drive the concurrent CS and ARF mortality burden. It was found that AMI was the most frequent specific underlying etiology, accounting for 10,135 deaths (20.7% of the overall cohort). Notably, the AAMR for AMI-related CS and ARF increased over twofold from 1999 (2.8) to 2023 (6.4), with rates rising sharply between 2018 and 2023 (APC: 18.91*; 95% CI: 12.87 to 31.92). HF and cardiac arrhythmias accounted for 2,062 (4.2%) and 991 (2.0%) deaths, respectively. Though AMI remained the dominant specific etiology, HF-associated deaths showed a starker relative acceleration in recent years, with AAMR surging from 0.2 in 2008 to 2.6 in 2023. This was similarly driven by a significant recent spike from 2018 to 2023 (APC: 27.17*; 95% CI: 22.41 to 38.29). Further, the reliable estimates for arrhythmia-induced cases revealed a significant increase in rates from 2015 to 2023 (APC: 28.05*; 95% CI: 21.61 to 48.85). However, the remaining deaths were attributed to broader or non-specific underlying cardiovascular and systemic etiologies, reflecting standard variations in death certificate coding practices (Supplemental Tables 1, 3, 10).

## Discussion

Using the CDC WONDER database, our study examined changes in CS and ARF-associated mortality among people aged ≥ 45 years in the U.S. during a 25-year period (1999–2023). This analysis shows a significant and steady rise in age-adjusted mortality during the study period, with a noticeable acceleration in recent years. Males, NH African Americans, older age groups, and populations living in the Southern and Western census regions, as well as non-metropolitan areas, were found to have a disproportionately higher mortality burden than other demographic and geographic subgroups.

A persistent rise in age-adjusted mortality linked to CS and ARF was noted in this national analysis, with a particularly noticeable increase in recent years. From 7.0 per million in 1999 to 43.5 per million in 2023, the total AAMR more than doubled. The first part of this pattern was quite stable until 2005. After that, there was a steady increasing trend until 2019 (APC 7.78%), and then there was a noticeable acceleration between 2019 and 2023 (APC 24.16%). This divergence in mortality trends in recent years can possibly be attributed to the variability in CS clinical profiles, especially the rising prevalence of chronic HF. Though previous population-based analyses showed reductions in overall AMI-related mortality [22], we found a different pattern for our highly specific cardiorenal cohort, where CS and ARF AAMR related to AMI increased from 1999 to 2023 with a sharp, significant acceleration from 2018 onward (APC 18.91%). Likewise, HF-induced cases experienced an even steeper relative increase over the same recent timeframe (APC 27.17%). Therefore, rather than a decrease in AMI offsetting a rise in HF, our data demonstrates that both etiologies are concurrently driving the escalating mortality burden in this specific dual-organ failure population. Furthermore, the most recent increase’s temporal clustering aligns with the COVID-19 pandemic period, which was marked by widespread reports of disruptions in the provision of cardiovascular care, delayed healthcare utilization, myocardial involvement linked to SARS-CoV-2 infection, and strain on the healthcare system [23, 24]. Prior research has identified the co-occurrence of ARF and CS as a high-risk cardiorenal phenotype, with ARF reported in 13–35% of CS patients and a continuously higher observed mortality in this cohort [25]. However, it should be noted that this is hypothetical and cannot be supported by our data.

Males consistently showed AAMRs that were nearly twice as high as those of females. This pattern seems contradictory, given the established literature showing higher short-term mortality in women once CS develops. This result highlights a basic difference between incidence and case-fatality rates: females who develop CS have 10–35% higher case-fatality rates after controlling for confounders, whereas males have significantly higher population-level mortality rates due to their markedly elevated incidence of CS across all age groups [26, 27]. Men have higher baseline risks from ischemic heart disease (IHD), smoking, and earlier onset of atherosclerotic disease, while women usually present with CS about ten years later than men and are more likely to have non-ischemic etiologies such as HF with preserved ejection fraction, Takotsubo syndrome, and myocarditis. These sex differences in cardiovascular risk factor profiles could hypothetically account for the higher incidence in males, but this cannot be supported by our analysis [28, 29]. The study’s temporal patterns show significant evolving trends: female mortality increased more gradually but showed an even steeper acceleration after 2019, while male mortality showed an early mild decline through 2005, followed by sharp, sustained increases. With women now surpassing men in obesity rates and experiencing increasingly similar prevalence of diabetes, hypertension, and smoking at older ages, this sharper recent rise in women is especially concerning and may reflect several convergent factors, including the closing gender gap in exposure to cardiometabolic risk factors [30]. The accelerated mortality trends seen after 2019 could also hypothetically be related to the COVID-19 pandemic’s disproportionate effect on women with CS, as well as ongoing treatment disparities, where women are still much less likely to receive timely revascularization, mechanical circulatory support, and invasive cardiac interventions [31].

Our results showed that NH African Americans exhibited the highest AAMRs, followed by Hispanics and NH Whites. These results are consistent with earlier population-based research showing that NH African American individuals consistently have a per capita burden of cardiorenal-related mortality that is roughly double that of NH Whites [32]. The temporal trends across racial and ethnic groups showed that NH Whites experienced an initial non-significant decline in mortality between 1999 and 2005. Conversely, NH African Americans and Hispanics experienced non-significant increases during their respective early study periods. Following these initial phases, all racial and ethnic groups demonstrated steady increases in mortality that significantly accelerated after 2019. These trends align with recent reports showing that NH African American populations have experienced a faster increase in CS mortality since 2009, especially for HF-related shock, with NH African American mortality rates rising faster than NH White mortality rates [33]. Studies that contend with demographic, comorbidities, and process items have determined that NH African American patients undergoing cardiovascular procedures present with an increased odds of developing ARF by 50% to 80% compared to NH White patients [34]. This finding remains constant across all baseline kidney function levels and could theoretically be attributed to various reasons. For example, diabetes mellitus and other risk factors for cardiovascular disease are present more often among NH African American and Hispanic patients suffering from CS, which can be explained by the fact that these groups tend to be younger and less likely to have insurance [35]. Geographic variables that may contribute to this disparity were based on discriminatory practices such as redlining, and racial residential segregation has led to lower rates of preventive care usage, increased risk of exposure to environmental pollutants, reduced accessibility to healthy foods, and limited access to healthcare facilities for residents of these neighborhoods [36]. Further genetic and epigenetic mechanisms play a major role as the APOL1 gene variants are frequent in populations of African descent [37]. These variants possess a high risk of kidney disease and adverse renal outcomes in these individuals and exert their effects independent of diabetic or hypertensive status [38]. Epigenetic modifications like DNA methylation, which increase cardiovascular and renal aging, can further be induced as a cumulative effect of persistent psychosocial stress, which is frequently termed weathering or allostatic load [39, 40]. Therefore, after the adjustment of income quartile and healthcare access, NH African Americans tend to have a higher baseline risk of adverse cardiorenal outcomes like concurrent CS and ARF than NH White populations.

Our analysis found that individuals aged ≥ 65 had a six-fold higher overall AAMR than individuals between the ages of 45 and 64. This illustrates the documented link between CS mortality and aging [41]. These results are consistent with a recent AHA statement showing that age is a non-modifiable risk factor for death in CS, with short-term mortality rates of 30% to 50% that gradually rise with age in all Society for Cardiovascular Angiography and Interventions (SCAI) shock severity stages [42]. Following CS, older persons have much greater short-term and long-term mortality rates; in certain trials of patients over 75, 30-day mortality rates exceeded 60% [43]. According to recent prospective registry data from the FRENSHOCK study, Patients of 75 years of age and older with CS had more than twice the risk of 1-month mortality (adjusted HR 2.5) and 1-year mortality (adjusted HR 2.01) when compared to younger patients [44]. Overall, different but steadily deteriorating trajectories were shown by age-specific temporal patterns. The number of middle-aged individuals increased steadily throughout the research period, with a slight increase in previous years and a significant acceleration in the most recent decade. Elderly individuals, on the other hand, showed a pattern that was initially steady and then sharply increasing towards the conclusion of the observation period. These trends align with CDC WONDER database analyses showing that mortality from CS has been rising faster among individuals under 65 since 2009, especially for shock related to HF, while older adults continue to have the highest absolute mortality burden [22]. The accelerated increase in mortality among middle-aged adults may be associated with a higher burden of cardiometabolic risk factors, such as obesity, diabetes, and hypertension, alongside the growing contribution of HF-related CS, but this cannot be confirmed in our analysis.

Our results reported that the South and West census regions had the highest mortality rates. These results are consistent with the CDC WONDER database analysis, which indicates that the South has the highest burden of CS and cardiorenal-related deaths [32]. Multicenter cohort studies further say that regional disparities in outcomes are associated with differences in healthcare infrastructure, access to advanced cardiac and renal care, and population health characteristics [45]. For instance, the South and Northeast have lower rates of coronary angiography, percutaneous coronary intervention, and mechanical circulatory support, which could be associated with greater death rates in these areas, although this cannot be confirmed by our analysis [33]. Urban-rural stratification further demonstrated higher overall AAMRs in non-metropolitan areas when compared to metropolitan areas. This pattern persisted throughout the study period; non-metropolitan areas continued to exhibit a higher proportional burden of deaths. These results are in line with recent national statistics showing that age-standardized mortality rates for ARF and cardiorenal diseases are higher in rural counties than in urban counties, with the difference growing over time, especially after 2010 [46]. Even after controlling for clinical and demographic variables, patients who arrive at rural hospitals with AMI or HF had greater short- and long-term mortality than those in urban settings, according to meta-analyses and large-scale research [47, 48]. The research has linked the higher death rate in non-metropolitan locations to variations in healthcare infrastructure, restricted access to advanced cardiac and renal therapy, longer transfer times, and lower rates of procedures guided by guidelines [46]. The concentration of high mortality rates in particular states and areas emphasizes the importance of the implementation of public health initiatives and enhancing the healthcare system in order to alleviate these enduring regional disparities.

### Limitations

This study has several limitations. Firstly, ICD coding practices can vary regionally, by time period, and certifying authority, which may introduce misclassification bias as mortality estimates can possibly be over- or underestimated, limiting the reliability. Secondly, the database lacks precise diagnosis timelines, which limits the identification of the clinical temporal sequence of ARF and CS on an individual patient basis. Further, the casual interpretation cannot be implied to CS and ARF being co-listed on the death certificates. However, our sensitivity analysis, which showed the same mortality curves even when ARF was explicitly coded as the underlying cause, reinforces the reliability of our population-level trends irrespective of which organ system failed first. Thirdly, the ecological nature of the study design made it impossible to assess individual-level clinical and behavioral risk factors such as comorbidity burden, severity of cardiac dysfunction, stage of renal impairment, treatment strategies, healthcare utilization, and socioeconomic factors. Further evolving diagnostic procedures, disease awareness, and variability in clinical definitions of ARF and CS over the 25-year study window might have influenced the observed temporal trends. Fifthly, we excluded Asian and Native American populations from our analysis because of data suppression and low mortality counts, which could compromise the statistical validity of joinpoint regression models and limit the meaningful trend analysis for racial and ethnic subgroups. Moreover, our etiological sub-analysis might be subjected to multiple-cause of death reporting limitations, as we successfully isolated AMI, HF, and arrhythmias as underlying causes, making 27% of the overall cohort, but the remaining deaths were coded under broader systemic categories. Lastly, a continuous joinpoint trend analysis for HF and arrhythmias was restricted to 2008–2023 and 2015–2023, respectively, due to low absolute death counts (*n* < 20) in earlier years that led to data suppression. Lastly, our findings are U.S. specific and may not be generalized to countries with diverse healthcare systems and demographics.

## Conclusions

Among U.S. adults aged ≥ 45 years over 25, our analysis reveals notable temporal patterns and enduring demographic and regional differences in mortality associated with concurrent CS and ARF. While AAMRs increased substantially, males consistently exhibited higher mortality than females. AAMRs showed a higher mortality burden among NH African Americans, older adults, and inhabitants of non-urban regions, even though absolute death counts were greatest among NH White individuals and metropolitan populations. In addition to significant state-level heterogeneity, the West and South had the highest mortality rates, while the Northeast had lower rates. These findings demonstrate sustained heterogeneity in mortality patterns over time and across population groups in the U.S. 

## Supplementary Information

Supplementary Material 1.

## Data Availability

The datasets generated during and/or analyzed during the current study are publicly available.
