# Supplementary material for: Cardiogenic shock and acute renal failure associated mortality trends in the United States: a retrospective analysis of death records from 1999 to 2023
Source: BMC Cardiovasc Disord. 2026 Mar 4;26:375. doi: 10.1186/s12872-026-05698-9 (PMC13130764; doi:10.1186/s12872-026-05698-9)
Supplement: Supplementary file 1 — Supplementary Material 1. [file 12872_2026_5698_MOESM1_ESM.docx]

**SUPPLEMENTARY FILE GLOSSARY**

**Supplemental Table 1.** Absolute number of Cardiogenic Shock and Acute renal failure-related deaths and percent total deaths among older adults stratified by overall, gender, race/ ethnicity, place of death, urbanization, and regions in the United States, 1999-2023.

**Supplementary Figure 1.** Percent total deaths of Cardiogenic Shock and Acute renal failure by place of death among older adults in the United States, 1999 to 2023.

**Supplemental Table 2.** Overall and sex-stratified Cardiogenic Shock and Acute renal failure-related age-adjusted mortality rates per 1,000,000 among adults in the United States, 1999 to 2023.

**Supplementary Figure 2.** Multiple Cardiogenic Shock and Acute renal failure-related age-adjusted mortality rates per 1,000,000, stratified by states among adults in the United States, 1999 to 2020

**Supplemental Table 3.** Annual percent change (APC) of Cardiogenic Shock and Acute renal failure-related age-adjusted mortality rates per 1,000,000 among adults in the United States, 1999 to 2023.

**Supplemental Table 4.** Sensitivity analysis of Acute Renal Failure as the underlying cause of death: age-adjusted mortality rates per 1,000,000 among adults in the United States, 1999 to 2023.

**Supplemental Table 5.** Race/ Ethnicity stratified Cardiogenic Shock and Acute renal failure-related age-adjusted mortality rates per 1,000,000 among adults in the United States, 1999 to 2023.

**Supplemental Table 6.** Age group-stratified Cardiogenic Shock and Acute renal failure-related age-adjusted mortality rates per 1,000,000 among adults in the United States, 1999 to 2023.

**Supplemental Table 7.** Urbanization stratified Cardiogenic Shock and Acute renal failure-Related age-adjusted mortality rates per 1,000,000 among adults in the United States from 1999 to 2020.

**Supplemental Table 8.** Region-stratified Cardiogenic Shock and Acute renal failure-related age-adjusted mortality rates per 1,000,000 among adults in the United States, 1999 to 2023.

**Supplemental Table 9.** State-stratified Cardiogenic Shock and Acute renal failure-related age-adjusted mortality rates per 1,000,000 and their respective percentiles among adults in the United States, 1999 to 2020.

**Supplemental Table 10.** Etiological subclassification of Cardiogenic Shock and Acute Renal Failure-related age-adjusted mortality rates per 1,000,000 among adults in the United States, 1999 to 2023.

| **Variables** | **Deaths** | **% of Total Deaths** |
| --- | --- | --- |
| Overall CS and ARF | 48926 | 100.00 |
| **Sensitivity Analysis** | | |
| ARF as UCD | 7872 | 16.09 |
| **Gender** | | |
| Female | 19459 | 39.77 |
| Male | 29467 | 60.23 |
| **Race/Ethnicity** | | |
| NH African American | 6635 | 13.56 |
| NH White | 35520 | 72.60 |
| Hispanic | 4240 | 8.67 |
| **Place of Deaths** | | |
| Medical Facilities | 47447 | 96.98 |
| Decedent's home | 357 | 0.73 |
| Hospice facility | 468 | 0.95 |
| Nursing home/long term care | 396 | 0.81 |
| Other | 164 | 0.33 |
| **Urbanization** | | |
| Metropolitan Areas | 25127 | 81.02 |
| Non-Metropolitan Areas | 5886 | 18.98 |
| **Regions** | | |
| Northeast | 7104 | 14.52 |
| Midwest | 9627 | 19.68 |
| South | 19642 | 40.15 |
| West | 12553 | 25.66 |
| **Age-groups** | | |
| Older Adults | 11197 | 22.89 |
| Middle aged Adults | 37729 | 77.11 |
| **Etiological subclassification** | | |
| AMI as UCD | 10135 | 20.71 |
| HF as UCD | 2062 | 4.21 |
| Cardiac Arrhythmia as UCD | 991 | 2.03 |
| ARF = Acute Renal Failure; UCD = Underlying Cause of Death; NH = Non-Hispanic; Older Adults = 65-85+ years old, Middle-aged Adults= 45-64 years old; AMI = Acute Myocardial Infarction; HF = Heart Failure | | |

**Supplemental Table 1.** Absolute number of Cardiogenic Shock and Acute renal failure-related deaths and percent total deaths among adults stratified by overall, gender, race/ ethnicity, place of death, urbanization, and regions in the United States, 1999-2023.

**Supplementary Figure 1.** Percent total deaths of Cardiogenic Shock and Acute renal failure by place of death among adults in the United States, 1999 to 2023.

| **Age-Adjusted Rate /1,000,000 (95% CI)** | | | |
| --- | --- | --- | --- |
| **Year** | **Overall** | **Male** | **Female** |
| 1999 | \| 7 (6.5–7.6) \| \| --- \| | \| 10.0 (8.9–11.0) \| \| --- \| | \| 5.1 (4.5–5.7) \| \| --- \| |
| 2000 | \| 7.3 (6.8–7.9) \| \| --- \| | \| 10.6 (9.6–11.7) \| \| --- \| | \| 5.2 (4.6–5.7) \| \| --- \| |
| 2001 | \| 7 (6.5–7.5) \| \| --- \| | \| 10.0 (9.0–11.0) \| \| --- \| | \| 5.1 (4.5–5.6) \| \| --- \| |
| 2002 | \| 7.2 (6.7–7.7) \| \| --- \| | \| 9.2 (8.3–10.2) \| \| --- \| | \| 5.8 (5.2–6.4) \| \| --- \| |
| 2003 | \| 7.2 (6.7–7.8) \| \| --- \| | \| 9.6 (8.7–10.6) \| \| --- \| | \| 5.6 (5.0–6.2) \| \| --- \| |
| 2004 | \| 7.1 (6.5–7.6) \| \| --- \| | \| 9.6 (8.7–10.6) \| \| --- \| | \| 5.4 (4.8–6.0) \| \| --- \| |
| 2005 | \| 7 (6.5–7.5) \| \| --- \| | \| 9.5 (8.6–10.5) \| \| --- \| | \| 5.2 (4.7–5.8) \| \| --- \| |
| 2006 | \| 7.6 (7.0–8.1) \| \| --- \| | \| 10.4 (9.4–11.3) \| \| --- \| | \| 5.6 (5.1–6.2) \| \| --- \| |
| 2007 | \| 8.2 (7.6–8.7) \| \| --- \| | \| 10.8 (9.8–11.8) \| \| --- \| | \| 6.2 (5.6–6.9) \| \| --- \| |
| 2008 | \| 9.2 (8.6–9.8) \| \| --- \| | \| 12.7 (11.6–13.7) \| \| --- \| | \| 6.7 (6.0–7.3) \| \| --- \| |
| 2009 | \| 9.6 (9.0–10.2) \| \| --- \| | \| 13.1 (12.1–14.2) \| \| --- \| | \| 7.1 (6.4–7.7) \| \| --- \| |
| 2010 | \| 10.7 (10.1–11.3) \| \| --- \| | \| 14.5 (13.4–15.6) \| \| --- \| | \| 7.9 (7.2–8.6) \| \| --- \| |
| 2011 | \| 11.2 (10.6–11.8) \| \| --- \| | \| 14.8 (13.8–15.9) \| \| --- \| | \| 8.4 (7.7–9.1) \| \| --- \| |
| 2012 | \| 11.6 (11.0–12.2) \| \| --- \| | \| 16.2 (15.1–17.3) \| \| --- \| | \| 8.1 (7.4–8.7) \| \| --- \| |
| 2013 | \| 11.9 (11.3–12.5) \| \| --- \| | \| 16.3 (15.2–17.4) \| \| --- \| | \| 8.6 (7.9–9.3) \| \| --- \| |
| 2014 | \| 12.5 (11.9–13.1) \| \| --- \| | \| 17.2 (16.1–18.3) \| \| --- \| | \| 8.9 (8.2–9.6) \| \| --- \| |
| 2015 | \| 14.5 (13.9–15.2) \| \| --- \| | \| 20.0 (18.8–21.2) \| \| --- \| | \| 10.4 (9.7–11.2) \| \| --- \| |
| 2016 | \| 17.0 (16.3–17.7) \| \| --- \| | \| 24.4 (23.1–25.6) \| \| --- \| | \| 11.1 (10.4–11.9) \| \| --- \| |
| 2017 | \| 17.5 (16.8–18.3) \| \| --- \| | \| 25.0 (23.7–26.3) \| \| --- \| | \| 11.7 (10.9–12.5) \| \| --- \| |
| 2018 | \| 18.8 (18.1–19.6) \| \| --- \| | \| 26.7 (25.4–28.0) \| \| --- \| | \| 12.4 (11.6–13.2) \| \| --- \| |
| 2019 | \| 20.0 (19.2–20.7) \| \| --- \| | \| 28.2 (26.9–29.5) \| \| --- \| | \| 13.4 (12.6–14.2) \| \| --- \| |
| 2020 | \| 21.2 (20.4–22.0) \| \| --- \| | \| 30.1 (28.7–31.4) \| \| --- \| | \| 13.9 (13.1–14.7) \| \| --- \| |
| 2021 | \| 33.7 (32.7–34.7) \| \| --- \| | \| 45.2 (43.6–46.9) \| \| --- \| | \| 24.1 (23.0–25.2) \| \| --- \| |
| 2022 | \| 42.8 (41.7–43.8) \| \| --- \| | \| 58.0 (56.2–59.9) \| \| --- \| | \| 30.5 (29.3–31.8) \| \| --- \| |
| 2023 | \| 43.5 (42.4–44.5) \| \| --- \| | \| 59.3 (57.5–61.2) \| \| --- \| | \| 30.6 (29.4–31.8) \| \| --- \| |
| **Total** | **14.9 (114.2–15.5)** | \| 20.5 (19.3–21.6) \| \| --- \| | 10.5 (9.8–11.3) |
| CI: Confidence Interval | | | |

**Supplemental Table 2.** Overall and sex-stratified Cardiogenic Shock and Acute renal failure-related age-adjusted mortality rates per 1,000,000 among adults in the United States, 1999 to 2023.


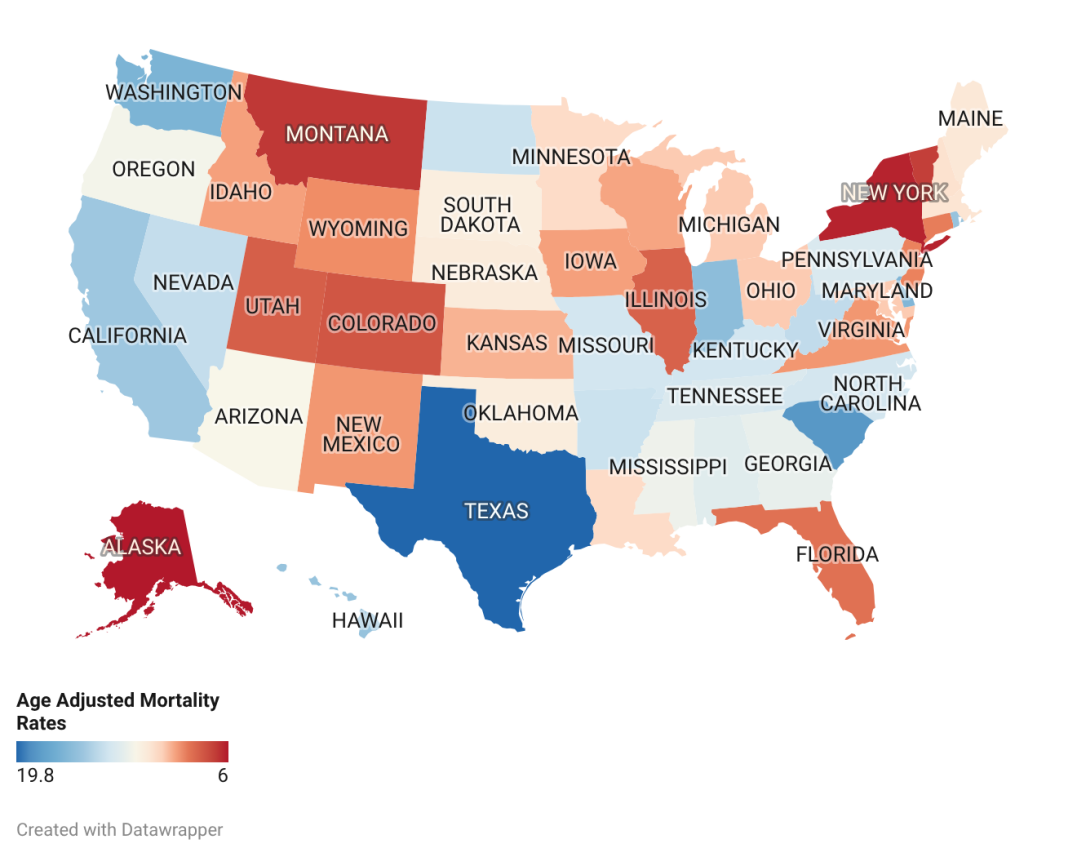


**Supplementary Figure 2.** Cardiogenic Shock and Acute renal failure-related age-adjusted mortality rates per 1,000,000, stratified by states among adults in the United States, 1999 to 2020.

| **Year Interval** | **APC (95% CI)** |
| --- | --- |
| **Overall** | |
| 1999-2005 | -0.34 (-17.28 to 5.68) |
| 2005-2019 | 7.78* (6.20 to 12.36) |
| 2019-2023 | 24.16* (19.47 to 33.06) |
| **Sensitivity Analysis (ARF as UCD)** | |
| 1999-2019 | 6.4* (4.4 - 7.6) |
| 2019-2023 | 16.1* (9.2 - 30.5) |
| **Gender** | |
| **Female** | |
| 1999-2019 | 5.56* (4.28 to 6.83) |
| 2019-2023 | 28.06* (20.98 to 40.60) |
| **Male** | |
| 1999-2005 | -1.32 (-16.66 to 4.10) |
| 2005-2019 | 8.17* (6.66 to 10.70) |
| 2019-2023 | 22.94* (18.29 to 31.41) |
| **Race** | |
| **NH African American** | |
| 1999-2012 | 4.69 (-9.56 to 31.89) |
| 2012-2019 | 10.12* (1.81 to 19.04) |
| 2019-2023 | 24.22* (17.47 to 36.44) |
| **NH White** | |
| 1999-2005 | -0.19 (-16.86 to 5.82) |
| 2005-2019 | 7.39* (5.55 to 13.13) |
| 2019-2023 | 25.13* (19.61 to 38.47) |
| **Hispanic** | |
| 2000-2013 | 3.85 (-4.83 to 7.89) |
| 2013-2023 | 14.21* (11.84 to 21.10) |
| **Urbanization** | |
| **Metropolitan** | |
| 1999-2005 | 0.00 (-1.68 to 1.28) |
| 2005-2010 | 8.40* (7.29 to 11.28) |
| 2010-2013 | 2.01* (0.60 to 3.89) |
| 2013-2016 | 13.55* (11.35 to 15.02) |
| 2016-2020 | 5.52* (4.25 to 6.42) |
| **Non-Metropolitan** | |
| 1999-2004 | -4.47 ( -13.54 to 0.59) |
| 2004-2020 | 8.75* (8.10 to 9.72) |
| **Age Groups** | |
| **Middle-aged Adults** | |
| 1999-2014 | 8.17*(2.70 to 11.27) |
| 2014-2013 | 18.4*(16.04 to 24.17) |
| **Older adults** | |
| 1999-2005 | -0.17(-14.47 to 4.89) |
| 2005-2019 | 6.60*(5.17 to 11.77) |
| 2019-2023 | 24.33*(19.51to 33.76) |
| **Etiological subclassification** | |
| **AMI as UCD** | |
| 1999-2018 | 1.33 (-0.09 - 2.56) |
| 2018-2023 | 18.91* (12.87 - 31.92) |
| **HF as UCD** | |
| 2008-2018 | 14.38 (-20.46 - 22.97) |
| 2018-2023 | 27.17* (22.41 - 38.29) |
| **Cardiac Arrhythmia as UCD** | |
| 2015-2023 | 28.05* (21.61 - 48.85) |
| Middle-aged Adults = 45-64 years; Older adults = 65-85+ years; NH = Non-Hispanic; APC = Annual Percent Change; AMI = Acute Myocardial Infarction; HF = Heart Failure; UCD = Underlying Cause of Death; CI = Confidence Interval | |

**Supplemental Table 3.** Annual percent change (APC) of Cardiogenic Shock and Acute renal failure-related age-adjusted mortality rates per 1,000,000 among adults in the United States, 1999 to 2023

| **Sensitivity Analysis** | |  |
| --- | --- | --- |
| **Year** | **Age-Adjusted Rate /1,000,000 (95% CI) (when ARF is the Underlying Cause)** |  |
| 1999 | 1 (0.8 - 1.2) |  |
| 2000 | 1.3 (1 - 1.5) |  |
| 2001 | 1 (0.8 - 1.2) |  |
| 2002 | 1.3 (1.1 - 1.5) |  |
| 2003 | 1.2 (0.9 - 1.4) |  |
| 2004 | 1.1 (0.9 - 1.3) |  |
| 2005 | 1.3 (1.1 - 1.5) |  |
| 2006 | 1.5 (1.3 - 1.7) |  |
| 2007 | 1.5 (1.2 - 1.7) |  |
| 2008 | 1.8 (1.5 - 2) |  |
| 2009 | 1.6 (1.4 - 1.9) |  |
| 2010 | 1.9 (1.6 - 2.1) |  |
| 2011 | 2.2 (1.9 - 2.5) |  |
| 2012 | 2.3 (2.1 - 2.6) |  |
| 2013 | 1.9 (1.7 - 2.2) |  |
| 2014 | 2.4 (2.1 - 2.7) |  |
| 2015 | 2.7 (2.4 - 2.9) |  |
| 2016 | 3 (2.8 - 3.3) |  |
| 2017 | 3.2 (2.9 - 3.5) |  |
| 2018 | 3.2 (2.9 - 3.5) |  |
| 2019 | 3.5 (3.2 - 3.8) |  |
| 2020 | 3.5 (3.2 - 3.8) |  |
| 2021 | 4.5 (4.1 - 4.8) |  |
| 2022 | 6 (5.6 - 6.4) |  |
| 2023 | 5.6 (5.3 - 6) |  |
| **Total** | 2.4 (2.2 - 2.7) |  |
| NH: Non-Hispanic; CI: Confidence Interval | |  |

**Supplemental Table 4.** Sensitivity analysis of Acute Renal Failure as the underlying cause of death: age-adjusted mortality rates per 1,000,000 among adults in the United States, 1999 to 2023.

| **Age-Adjusted Rate /1,000,000 (95% CI)** | | | |
| --- | --- | --- | --- |
| **Year** | **NH African American** | **NH White** | **Hispanic** |
| 1999 | \| 9.4 (7.3–11.8) \| \| --- \| | \| 6.9 (6.3–7.4) \| \| --- \| | Unreliable |
| 2000 | \| 8.9 (7.0–11.2) \| \| --- \| | \| 7.0 (6.4–7.6) \| \| --- \| | \| 9.1 (6.5–12.5) \| \| --- \| |
| 2001 | \| 8.5 (6.6–10.8) \| \| --- \| | \| 6.8 (6.3–7.4) \| \| --- \| | \| 6.7 (4.6–9.4) \| \| --- \| |
| 2002 | \| 10.2 (8.1–12.7) \| \| --- \| | \| 6.9 (6.4–7.5) \| \| --- \| | \| 4.4 (2.8–6.7) \| \| --- \| |
| 2003 | \| 8.6 (6.7–10.8) \| \| --- \| | \| 7.0 (6.4–7.5) \| \| --- \| | \| 8.8 (6.4–11.8) \| \| --- \| |
| 2004 | \| 9.4 (7.5–11.8) \| \| --- \| | \| 6.8 (6.3–7.4) \| \| --- \| | \| 7.5 (5.4–10.1) \| \| --- \| |
| 2005 | \| 9.4 (7.5–11.7) \| \| --- \| | \| 6.7 (6.2–7.3) \| \| --- \| | \| 6.7 (4.7–9.1) \| \| --- \| |
| 2006 | \| 9.8 (7.9–12.1) \| \| --- \| | \| 7.2 (6.7–7.8) \| \| --- \| | \| 7.8 (5.7–10.3) \| \| --- \| |
| 2007 | \| 12.4 (10.1–14.7) \| \| --- \| | \| 7.7 (7.1–8.3) \| \| --- \| | \| 7.8 (5.7–10.3) \| \| --- \| |
| 2008 | \| 14.2 (11.8–16.6) \| \| --- \| | \| 8.7 (8.1–9.3) \| \| --- \| | \| 8.8 (6.7–11.3) \| \| --- \| |
| 2009 | \| 12.7 (10.5–14.9) \| \| --- \| | \| 9.1 (8.5–9.7) \| \| --- \| | \| 9.9 (7.7–12.5) \| \| --- \| |
| 2010 | \| 12.8 (10.6–15.0) \| \| --- \| | \| 10.5 (9.9–11.2) \| \| --- \| | \| 9.9 (7.8–12.4) \| \| --- \| |
| 2011 | \| 14.3 (12.0–16.6) \| \| --- \| | \| 11.0 (10.3–11.6) \| \| --- \| | \| 10.1 (8.0–12.5) \| \| --- \| |
| 2012 | \| 15.7 (13.3–18.1) \| \| --- \| | \| 11.2 (10.5–11.9) \| \| --- \| | \| 10.9 (8.8–13.3) \| \| --- \| |
| 2013 | \| 16.5 (14.1–18.9) \| \| --- \| | \| 11.3 (10.6–12.0) \| \| --- \| | \| 11.5 (9.3–13.7) \| \| --- \| |
| 2014 | \| 16.1 (13.8–18.5) \| \| --- \| | \| 11.8 (11.1–12.5) \| \| --- \| | \| 13.4 (11.1–15.7) \| \| --- \| |
| 2015 | \| 19.2 (16.7–21.7) \| \| --- \| | \| 13.9 (13.1–14.6) \| \| --- \| | \| 15.3 (12.9–17.6) \| \| --- \| |
| 2016 | \| 24.1 (21.4–26.9) \| \| --- \| | \| 16.5 (15.7–17.3) \| \| --- \| | \| 12.8 (10.7–14.9) \| \| --- \| |
| 2017 | \| 22.1 (19.5–24.6) \| \| --- \| | \| 16.4 (15.6–17.2) \| \| --- \| | \| 20.7 (18.1–23.3) \| \| --- \| |
| 2018 | \| 28.7 (25.8–31.6) \| \| --- \| | \| 17.3 (16.5–18.1) \| \| --- \| | \| 20.3 (17.8–22.8) \| \| --- \| |
| 2019 | \| 27.7 (24.9–30.5) \| \| --- \| | \| 18.9 (18.1–19.8) \| \| --- \| | \| 19.9 (17.5–22.3) \| \| --- \| |
| 2020 | \| 32.3 (29.3–35.2) \| \| --- \| | \| 19.2 (18.3–20.0) \| \| --- \| | \| 24.2 (21.6–26.9) \| \| --- \| |
| 2021 | \| 47.9 (44.2–51.5) \| \| --- \| | \| 31.8 (30.7–32.9) \| \| --- \| | \| 34.7 (31.6–37.8) \| \| --- \| |
| 2022 | \| 60.8 (56.8–64.9) \| \| --- \| | \| 41.0 (39.8–42.3) \| \| --- \| | \| 39.7 (36.4–42.9) \| \| --- \| |
| 2023 | \| 64.8 (60.6–69.0) \| \| --- \| | \| 41.8 (40.5–43.0) \| \| --- \| | \| 38.9 (35.8–42.1) \| \| --- \| |
| **Total** | \| 20.7 (18.2–23.3) \| \| --- \| | \| 14.1 (13.4–14.9) \| \| --- \| | \| 15.0 (12.7–17.6) \| \| --- \| |
| NH = Non-Hispanic; CI = Confidence Interval | | | |

**Supplemental Table 5.** Race/Ethnicity stratified Cardiogenic Shock and Acute renal failure-related age-adjusted mortality rates per 1,000,000 among adults in the United States, 1999 to 2023

| **Age-Adjusted Rate /1,000,000 (95% CI)** | | |
| --- | --- | --- |
| **Year** | **Middle-aged Adults** | **Older Adults** |
| 1999 | \| 1.4 (1.1 – 1.8) \| \| --- \| | \| 16.9 (15.6 – 18.3) \| \| --- \| |
| 2000 | \| 1.7 (1.4 – 2.1) \| \| --- \| | \| 17.2 (15.8 – 18.5) \| \| --- \| |
| 2001 | \| 1.5 (1.2 – 1.9) \| \| --- \| | \| 16.6 (15.3 – 18.0) \| \| --- \| |
| 2002 | \| 1.6 (1.3 – 1.9) \| \| --- \| | \| 17.0 (15.7 – 18.4) \| \| --- \| |
| 2003 | \| 1.6 (1.3 – 1.9) \| \| --- \| | \| 17.1 (15.7 – 18.4) \| \| --- \| |
| 2004 | \| 1.6 (1.3 – 1.9) \| \| --- \| | \| 16.6 (15.3 – 18.0) \| \| --- \| |
| 2005 | \| 1.7 (1.4 – 2.0) \| \| --- \| | \| 16.3 (15.0 – 17.6) \| \| --- \| |
| 2006 | \| 1.9 (1.6 – 2.2) \| \| --- \| | \| 17.5 (16.2 – 18.9) \| \| --- \| |
| 2007 | \| 1.8 (1.5 – 2.1) \| \| --- \| | \| 19.3 (17.9 – 20.7) \| \| --- \| |
| 2008 | \| 2.4 (2.1 – 2.8) \| \| --- \| | \| 21.1 (19.6 – 22.5) \| \| --- \| |
| 2009 | \| 2.4 (2.1 – 2.7) \| \| --- \| | \| 22.2 (20.8 – 23.7) \| \| --- \| |
| 2010 | \| 2.9 (2.6 – 3.3) \| \| --- \| | \| 24.4 (22.8 – 25.9) \| \| --- \| |
| 2011 | \| 3.2 (2.8 – 3.6) \| \| --- \| | \| 25.2 (23.7 – 26.7) \| \| --- \| |
| 2012 | \| 3.6 (3.2 – 4.0) \| \| --- \| | \| 25.5 (24.0 – 27.0) \| \| --- \| |
| 2013 | \| 3.8 (3.4 – 4.2) \| \| --- \| | \| 26.1 (24.6 – 27.6) \| \| --- \| |
| 2014 | \| 3.8 (3.4 – 4.2) \| \| --- \| | \| 27.7 (26.2 – 29.3) \| \| --- \| |
| 2015 | \| 4.7 (4.3 – 5.2) \| \| --- \| | \| 31.8 (30.1 – 33.4) \| \| --- \| |
| 2016 | \| 6.2 (5.7 – 6.8) \| \| --- \| | \| 35.8 (34.1 – 37.6) \| \| --- \| |
| 2017 | \| 6.4 (5.9 – 7.0) \| \| --- \| | \| 37.1 (35.4 – 38.8) \| \| --- \| |
| 2018 | \| 7.5 (6.9 – 8.1) \| \| --- \| | \| 38.7 (37.0 – 40.5) \| \| --- \| |
| 2019 | \| 7.9 (7.4 – 8.5) \| \| --- \| | \| 41.1 (39.4 – 42.9) \| \| --- \| |
| 2020 | \| 8.5 (7.9 – 9.1) \| \| --- \| | \| 43.5 (41.7 – 45.3) \| \| --- \| |
| 2021 | \| 14.0 (13.2 – 14.7) \| \| --- \| | \| 68.4 (66.1 – 70.7) \| \| --- \| |
| 2022 | \| 16.6 (15.8 – 17.5) \| \| --- \| | \| 88.7 (86.2 – 91.2) \| \| --- \| |
| 2023 | \| 17.1 (16.3 – 18.0) \| \| --- \| | \| 89.8 (87.3 – 92.3) \| \| --- \| |
| **Total** | \| 5.0 (4.6 – 5.5) \| \| --- \| | \| 32.1 (30.5 – 33.7) \| \| --- \| |
| Middle-aged Adults = 45-64 years, Older Adults = 65-85+ years; CI = Confidence Interval | | |

**Supplemental Table 6.** Age group-stratified Cardiogenic Shock and Acute renal failure-related age-adjusted mortality rates per 1,000,000 among adults in the United States, 1999 to 2023.

| **Age-Adjusted Rate /1,000,000 (95% CI)** | | |
| --- | --- | --- |
| **Year** | **Metropolitan** | **Non-Metropolitan** |
| 1999 | \| 6.8 (6.2–7.3) \| \| --- \| | 8.4 (7.1–9.7) |
| 2000 | \| 7.2 (6.6–7.8) \| \| --- \| | 7.8 (6.6–9.1) |
| 2001 | \| 7.0 (6.4–7.6) \| \| --- \| | 7.2 (6.0–8.4) |
| 2002 | \| 7.1 (6.5–7.6) \| \| --- \| | 7.7 (6.4–9.0) |
| 2003 | \| 7.4 (6.8–8.0) \| \| --- \| | 6.1 (5.0–7.3) |
| 2004 | \| 7.2 (6.6–7.7) \| \| --- \| | 6.7 (5.5–7.8) |
| 2005 | \| 6.9 (6.4–7.5) \| \| --- \| | 7.0 (5.9–8.2) |
| 2006 | \| 7.6 (7.0–8.2) \| \| --- \| | 7.4 (6.2–8.6) |
| 2007 | \| 8.0 (7.4–8.6) \| \| --- \| | 8.9 (7.6–10.2) |
| 2008 | \| 9.1 (8.5–9.8) \| \| --- \| | 9.5 (8.1–10.8) |
| 2009 | \| 9.6 (9.0–10.2) \| \| --- \| | 9.4 (8.1–10.7) |
| 2010 | \| 10.6 (9.9–11.2) \| \| --- \| | 11.5 (10.0–12.9) |
| 2011 | \| 10.9 (10.3–11.6) \| \| --- \| | 12.6 (11.1–14.1) |
| 2012 | \| 11.1 (10.5–11.8) \| \| --- \| | 13.5 (11.9–15.1) |
| 2013 | \| 11.5 (10.9–12.2) \| \| --- \| | 13.5 (11.9–15.0) |
| 2014 | \| 12.3 (11.6–13.0) \| \| --- \| | 13.2 (11.7–14.8) |
| 2015 | \| 14.1 (13.4–14.8) \| \| --- \| | 16.7 (15.0–18.4) |
| 2016 | \| 16.7 (15.9–17.4) \| \| --- \| | 18.7 (16.8–20.5) |
| 2017 | \| 17.4 (16.6–18.1) \| \| --- \| | 18.7 (16.9–20.5) |
| 2018 | \| 18.4 (17.7–19.2) \| \| --- \| | 20.9 (19.0–22.8) |
| 2019 | \| 19.3 (18.5–20.1) \| \| --- \| | 23.3 (21.3–25.3) |
| 2020 | \| 20.3 (19.5–21.1) \| \| --- \| | 25.6 (23.5–27.7) |
| **Total** | \| 11.8 (11.6–11.9) \| \| --- \| | 12.9 (12.6–13.2) |
| CI = Confidence Interval | | |

**Supplemental Table 7.** Urbanization stratified Cardiogenic Shock and Acute renal failure-Related age-adjusted mortality rates per 1,000,000 among adults in the United States from 1999 to 2020.

| **Age-Adjusted Rate /1,000,000 (95% CI)** | | | | |
| --- | --- | --- | --- | --- |
| **Year** | **Northeast** | **Midwest** | **South** | **West** |
| 1999 | \| 8.3 (7.0–9.5) \| \| --- \| | \| 7.1 (6.0–8.2) \| \| --- \| | \| 6.7 (5.8–7.5) \| \| --- \| | \| 6.3 (5.2–7.5) \| \| --- \| |
| 2000 | \| 7.9 (6.7–9.1) \| \| --- \| | \| 7.3 (6.2–8.4) \| \| --- \| | \| 7.9 (7.0–8.9) \| \| --- \| | \| 5.7 (4.6–6.8) \| \| --- \| |
| 2001 | \| 7.0 (5.9–8.1) \| \| --- \| | \| 7.5 (6.4–8.7) \| \| --- \| | \| 7.7 (6.7–8.6) \| \| --- \| | \| 5.2 (4.2–6.2) \| \| --- \| |
| 2002 | \| 7.5 (6.3–8.7) \| \| --- \| | \| 7.1 (6.0–8.1) \| \| --- \| | \| 7.3 (6.4–8.2) \| \| --- \| | \| 6.8 (5.7–8.0) \| \| --- \| |
| 2003 | \| 7.8 (6.6–9.0) \| \| --- \| | \| 6.9 (5.9–8.0) \| \| --- \| | \| 6.6 (5.8–7.5) \| \| --- \| | \| 8.0 (6.7–9.2) \| \| --- \| |
| 2004 | \| 6.5 (5.4–7.6) \| \| --- \| | \| 6.7 (5.7–7.8) \| \| --- \| | \| 7.1 (6.2–7.9) \| \| --- \| | \| 8.0 (6.8–9.3) \| \| --- \| |
| 2005 | \| 7.4 (6.2–8.5) \| \| --- \| | \| 5.5 (4.6–6.5) \| \| --- \| | \| 7.2 (6.4–8.1) \| \| --- \| | \| 7.7 (6.5–8.8) \| \| --- \| |
| 2006 | \| 8.4 (7.2–9.6) \| \| --- \| | \| 5.7 (4.7–6.6) \| \| --- \| | \| 8.2 (7.3–9.1) \| \| --- \| | \| 7.5 (6.4–8.7) \| \| --- \| |
| 2007 | \| 6.9 (5.8–8.0) \| \| --- \| | \| 7.0 (5.9–8.0) \| \| --- \| | \| 9.4 (8.4–10.4) \| \| --- \| | \| 8.6 (7.4–9.8) \| \| --- \| |
| 2008 | \| 9.1 (7.8–10.4) \| \| --- \| | \| 7.8 (6.8–8.9) \| \| --- \| | \| 9.3 (8.3–10.2) \| \| --- \| | \| 10.6 (9.3–12.0) \| \| --- \| |
| 2009 | \| 8.6 (7.4–9.8) \| \| --- \| | \| 9.0 (7.9–10.2) \| \| --- \| | \| 9.6 (8.6–10.5) \| \| --- \| | \| 11.1 (9.7–12.4) \| \| --- \| |
| 2010 | \| 9.2 (8.0–10.5) \| \| --- \| | \| 10.7 (9.4–11.9) \| \| --- \| | \| 10.3 (9.3–11.3) \| \| --- \| | \| 12.8 (11.4–14.2) \| \| --- \| |
| 2011 | \| 9.6 (8.3–10.9) \| \| --- \| | \| 9.8 (8.6–11.0) \| \| --- \| | \| 11.8 (10.8–12.8) \| \| --- \| | \| 13.0 (11.6–14.4) \| \| --- \| |
| 2012 | \| 9.5 (8.2–10.7) \| \| --- \| | \| 11.4 (10.1–12.7) \| \| --- \| | \| 12.0 (10.9–13.0) \| \| --- \| | \| 12.9 (11.5–14.3) \| \| --- \| |
| 2013 | \| 9.0 (7.8–10.3) \| \| --- \| | \| 11.3 (10.1–12.6) \| \| --- \| | \| 12.1 (11.1–13.1) \| \| --- \| | \| 14.6 (13.1–16.0) \| \| --- \| |
| 2014 | \| 9.3 (8.1–10.5) \| \| --- \| | \| 11.9 (10.6–13.2) \| \| --- \| | \| 13.7 (12.6–14.8) \| \| --- \| | \| 13.7 (12.3–15.1) \| \| --- \| |
| 2015 | \| 10.0 (8.7–11.2) \| \| --- \| | \| 12.4 (11.0–13.7) \| \| --- \| | \| 16.5 (15.4–17.7) \| \| --- \| | \| 17.1 (15.6–18.6) \| \| --- \| |
| 2016 | \| 11.5 (10.2–12.9) \| \| --- \| | \| 15.1 (13.6–16.5) \| \| --- \| | \| 19.1 (17.9–20.4) \| \| --- \| | \| 19.8 (18.2–21.5) \| \| --- \| |
| 2017 | \| 12.3 (10.9–13.6) \| \| --- \| | \| 15.4 (14.0–16.8) \| \| --- \| | \| 20.3 (19.0–21.5) \| \| --- \| | \| 19.6 (18.0–21.2) \| \| --- \| |
| 2018 | \| 13.2 (11.8–14.6) \| \| --- \| | \| 16.0 (14.6–17.5) \| \| --- \| | \| 22.0 (20.7–23.3) \| \| --- \| | \| 20.7 (19.1–22.3) \| \| --- \| |
| 2019 | \| 13.1 (11.7–14.5) \| \| --- \| | \| 18.9 (17.3–20.4) \| \| --- \| | \| 21.9 (20.7–23.2) \| \| --- \| | \| 23.3 (21.6–25.0) \| \| --- \| |
| 2020 | \| 13.7 (12.3–15.1) \| \| --- \| | \| 17.0 (15.5–18.4) \| \| --- \| | \| 23.8 (22.5–25.1) \| \| --- \| | \| 26.7 (24.9–28.4) \| \| --- \| |
| 2021 | \| 22.1 (20.3–23.9) \| \| --- \| | \| 29.8 (27.8–31.8) \| \| --- \| | \| 36.1 (34.4–37.7) \| \| --- \| | \| 42.7 (40.4–45.0) \| \| --- \| |
| 2022 | \| 29.2 (27.1–31.2) \| \| --- \| | \| 40.4 (38.1–42.6) \| \| --- \| | \| 45.4 (43.6–47.1) \| \| --- \| | \| 51.5 (49.1–54.0) \| \| --- \| |
| 2023 | \| 31.4 (29.3–33.5) \| \| --- \| | \| 38.5 (36.3–40.7) \| \| --- \| | \| 45.8 (44.0–47.5) \| \| --- \| | \| 54.0 (51.5–56.5) \| \| --- \| |
| **Total** | \| 11.5 (10.2–12.9) \| \| --- \| | \| 13.4 (12.1–14.8) \| \| --- \| | \| 15.9 (14.8–17.0) \| \| --- \| | \| 17.1 (15.6–18.6) \| \| --- \| |
| CI: Confidence Interval | | | | |

**Supplemental Table 8.** Region-stratified Cardiogenic Shock and Acute renal failure-related age-adjusted mortality rates per 1,000,000 among adults in the United States, 1999 to 2023.

| **State** | **Age-Adjusted Rate /1,000,000 (95% CI)** | **Percentile (%)** |
| --- | --- | --- |
| Alaska | \| 6.0 (3.5 – 9.5) \| \| --- \| | 0.00 |
| New York | \| 6.2 (5.8 – 6.6) \| \| --- \| | 2.00 |
| Montana | \| 6.7 (5.1 – 8.6) \| \| --- \| | 4.00 |
| Vermont | \| 6.9 (4.9 – 9.5) \| \| --- \| | 6.00 |
| Colorado | \| 7.6 (6.7 – 8.5) \| \| --- \| | 8.00 |
| Utah | \| 7.9 (6.5 – 9.3) \| \| --- \| | 10.00 |
| Illinois | \| 8.0 (7.5 – 8.5) \| \| --- \| | 12.00 |
| Florida | \| 8.5 (8.1 – 8.9) \| \| --- \| | 14.00 |
| Connecticut | \| 8.7 (7.7 – 9.7) \| \| --- \| | 16.00 |
| New Jersey | \| 8.8 (8.2 – 9.5) \| \| --- \| | 18.00 |
| Wyoming | \| 9.0 (6.4 – 12.2) \| \| --- \| | 20.00 |
| New Mexico | \| 9.2 (7.7 – 10.6) \| \| --- \| | 22.00 |
| Virginia | \| 9.2 (8.4 – 9.9) \| \| --- \| | 22.00 |
| Idaho | \| 9.4 (7.6 – 11.1) \| \| --- \| | 26.00 |
| Iowa | \| 9.4 (8.3 – 10.5) \| \| --- \| | 26.00 |
| Wisconsin | \| 9.5 (8.6 – 10.3) \| \| --- \| | 30.00 |
| Kansas | \| 9.8 (8.6 – 11.1) \| \| --- \| | 32.00 |
| Maryland | \| 10.2 (9.3 – 11.2) \| \| --- \| | 34.00 |
| Michigan | \| 10.2 (9.5 – 10.9) \| \| --- \| | 34.00 |
| Ohio | \| 10.2 (9.6 – 10.8) \| \| --- \| | 34.00 |
| Louisiana | \| 10.5 (9.4 – 11.5) \| \| --- \| | 40.00 |
| Minnesota | \| 10.5 (9.5 – 11.5) \| \| --- \| | 40.00 |
| New Hampshire | \| 10.8 (8.9 – 12.7) \| \| --- \| | 44.00 |
| Massachusetts | \| 11.0 (10.1 – 11.8) \| \| --- \| | 46.00 |
| Maine | \| 11.2 (9.4 – 13.0) \| \| --- \| | 48.00 |
| Nebraska | \| 11.4 (9.7 – 13.0) \| \| --- \| | 50.00 |
| Oklahoma | \| 11.5 (10.4 – 12.7) \| \| --- \| | 52.00 |
| District of Columbia | \| 11.6 (8.6 – 15.2) \| \| --- \| | 54.00 |
| South Dakota | \| 11.6 (9.3 – 14.3) \| \| --- \| | 54.00 |
| Arizona | \| 12.1 (11.1 – 13.0) \| \| --- \| | 58.00 |
| Oregon | \| 12.3 (11.1 – 13.4) \| \| --- \| | 60.00 |
| Mississippi | \| 12.5 (11.1 – 13.9) \| \| --- \| | 62.00 |
| Georgia | \| 12.7 (11.8 – 13.5) \| \| --- \| | 64.00 |
| Alabama | \| 13.0 (11.9 – 14.1) \| \| --- \| | 66.00 |
| Tennessee | \| 13.2 (12.2 – 14.2) \| \| --- \| | 68.00 |
| Pennsylvania | \| 13.3 (12.6 – 13.9) \| \| --- \| | 70.00 |
| North Carolina | \| 13.7 (12.9 – 14.5) \| \| --- \| | 72.00 |
| Kentucky | \| 13.8 (12.6 – 15.0) \| \| --- \| | 74.00 |
| Missouri | \| 13.8 (12.8 – 14.8) \| \| --- \| | 74.00 |
| Arkansas | \| 14.1 (12.6 – 15.5) \| \| --- \| | 78.00 |
| North Dakota | \| 14.1 (11.4 – 17.4) \| \| --- \| | 78.00 |
| Nevada | \| 14.3 (12.7 – 16.0) \| \| --- \| | 82.00 |
| West Virginia | \| 14.4 (12.7 – 16.2) \| \| --- \| | 84.00 |
| California | \| 15.4 (15.0 – 15.9) \| \| --- \| | 86.00 |
| Hawaii | \| 15.7 (13.5 – 18.0) \| \| --- \| | 88.00 |
| Rhode Island | \| 15.7 (13.2 – 18.1) \| \| --- \| | 88.00 |
| Indiana | \| 16.1 (15.0 – 17.1) \| \| --- \| | 92.00 |
| Delaware | \| 16.6 (13.8 – 19.5) \| \| --- \| | 94.00 |
| Washington | \| 16.8 (15.7 – 17.9) \| \| --- \| | 96.00 |
| South Carolina | \| 18.7 (17.4 – 20.1) \| \| --- \| | 98.00 |
| Texas | \| 19.8 (19.1 – 20.4) \| \| --- \| | 100.00 |
| CI = Confidence Interval | | |

**Supplemental Table 9.** State-stratified Cardiogenic Shock and Acute renal failure-related age-adjusted mortality rates per 1,000,000 and their respective percentiles among adults in the United States, 1999 to 2023.

| **Etiological Subclassification of CS and ARF (Age-Adjusted Rate /1,000,000; 95% CI)** | | | |
| --- | --- | --- | --- |
| **Year** | **Acute Myocardial Infarction** | **Heart Failure** | **Cardiac Arrhythmias** |
| 1999 | 2.8 (2.5 - 3.1) | Unreliable | Unreliable |
| 2000 | 2.5 (2.2 - 2.8) | Unreliable | Unreliable |
| 2001 | 2.7 (2.3 - 3) | Unreliable | Unreliable |
| 2002 | 2.5 (2.2 - 2.8) | Unreliable | Unreliable |
| 2003 | 2.6 (2.3 - 2.9) | Unreliable | Unreliable |
| 2004 | 2.4 (2.1 - 2.7) | Unreliable | Unreliable |
| 2005 | 2.3 (2.1 - 2.6) | Unreliable | Unreliable |
| 2006 | 2.2 (1.9 - 2.5) | Unreliable | Unreliable |
| 2007 | 2.4 (2.1 - 2.7) | \| Unreliable \| \| --- \| | Unreliable |
| 2008 | 2.6 (2.3 - 2.9) | 0.2 (0.1 - 0.3) | \| Unreliable \| \| --- \| |
| 2009 | 3 (2.7 - 3.3) | 0.3 (0.2 - 0.4) | Unreliable |
| 2010 | 3 (2.6 - 3.3) | 0.2 (0.1 - 0.3) | Unreliable |
| 2011 | 2.7 (2.4 - 3) | 0.3 (0.2 - 0.4) | Unreliable |
| 2012 | 2.8 (2.5 - 3.1) | 0.4 (0.3 - 0.5) | \| 10.9 (8.8–13.3) \| \| --- \| |
| 2013 | 2.9 (2.6 - 3.3) | 0.3 (0.2 - 0.4) | \| 11.5 (9.3–13.7) \| \| --- \| |
| 2014 | 2.8 (2.5 - 3.1) | 0.4 (0.3 - 0.5) | \| Unreliable \| \| --- \| |
| 2015 | 2.8 (2.5 - 3) | 0.7 (0.6 - 0.9) | \| 15.3 (12.9–17.6) \| \| --- \| |
| 2016 | 3.4 (3.1 - 3.7) | 0.6 (0.5 - 0.8) | \| 12.8 (10.7–14.9) \| \| --- \| |
| 2017 | 3 (2.7 - 3.3) | 0.7 (0.5 - 0.8) | \| 20.7 (18.1–23.3) \| \| --- \| |
| 2018 | 3.3 (3 - 3.6) | 0.8 (0.7 - 1) | \| 20.3 (17.8–22.8) \| \| --- \| |
| 2019 | 3.4 (3.1 - 3.8) | 1 (0.8 - 1.2) | \| 19.9 (17.5–22.3) \| \| --- \| |
| 2020 | 3.3 (3 - 3.6) | 1.2 (1.1 - 1.4) | \| 24.2 (21.6–26.9) \| \| --- \| |
| 2021 | 5.6 (5.3 - 6) | 1.8 (1.6 - 2) | \| 34.7 (31.6–37.8) \| \| --- \| |
| 2022 | 6.9 (6.5 - 7.3) | 2.4 (2.1 - 2.7) | \| 39.7 (36.4–42.9) \| \| --- \| |
| 2023 | 6.4 (6 - 6.9) | 2.6 (2.3 - 2.9) | \| 38.9 (35.8–42.1) \| \| --- \| |
| **Total** | 3.2 (2.9 - 3.5) | 0.9 (0.7 - 1) | \| 15.0 (12.7–17.6) \| \| --- \| |
| CS = Cardiogenic Shock; ARF = Acute Renal Failure; CI: Confidence Interval | | | |

**Supplemental Table 10.** Etiological subclassification of Cardiogenic Shock and Acute Renal Failure-related age-adjusted mortality rates per 1,000,000 among adults in the United States, 1999 to 2023.
